# Unusual symptoms reveal a rare splenic lymphoma: a case report of PS-DLBCL with neck and shoulder pain as the initial presentation

**DOI:** 10.3389/fonc.2025.1449966

**Published:** 2025-02-04

**Authors:** Shifu Hu, Yuanyuan Hao, Xiangyu Liu, Hanbo Liu

**Affiliations:** ^1^ Department of General Surgery, Tianjin Xiqing Hospital, Tianjin, China; ^2^ Department of Geriatrics, Tianjin Xiqing Hospital, Tianjin, China

**Keywords:** spontaneous splenic rupture, primary splenic diffuse large B-cell lymphoma, splenectomy, Kehr’s sign, case report

## Abstract

The rare occurrence of diffuse large B-cell lymphoma (DLBCL) limited to the spleen presents difficulties in diagnosis. A case report details a patient whose initial symptoms were neck and shoulder pain, subsequently diagnosed as spontaneous spleen rupture, a rare complication of primary splenic lymphoma. Following a splenectomy, the patient was confirmed to have primary splenic DLBCL and made a good postoperative recovery. This report highlights the diagnostic dilemmas in atypical presentations, where neck and shoulder pain may be the only early sign of a neglected spleen rupture, lacking typical symptoms of the underlying disease. In cases of patients who are not stable, the recommended first imaging method is a focused assessment using sonography for trauma (FAST), as CT scans have a high level of sensitivity. Treatment is determined by hemodynamic status, with conservative management for stable patients and surgical intervention for unstable patients. The importance of recognizing spontaneous spleen rupture as a critical yet uncommon possibility in acute abdominal cases, especially in instances of primary splenic DLBCL, is emphasized in the report.

## 1 Introduction

Spontaneous splenic rupture (SSR) is a rare and critical medical event, first documented by Rokitansky in 1861 and subsequently by Atkinson in 1874, representing only 1% of total spleen rupture cases (1). This condition can lead to hemodynamic instability, is challenging to diagnose, and can be life-threatening if not promptly identified. The incidence of SSR has historically been very low, but it is now understood as a secondary complication often stemming from underlying pathological conditions. SSR can be triggered by various factors, including infections, vasculitis, pancreatitis, hematological disorders, or tumors (2). It is imperative for healthcare providers to consider primary splenic diffuse large B-cell lymphoma (PS-DLBCL), an exceedingly rare subtype of primary splenic lymphoma accounting for less than 1% of cases, as a potential cause of SSR. The non-specific symptoms of SSR can lead to misdiagnosis, delaying crucial treatment. The pathophysiology of SSR remains unclear, and both SSR and PS-DLBCL are rare, with SSR occasionally being the first manifestation of PS-DLBCL, a scenario that is seldom documented. This case report presents a unique instance of SSR resulting from PS-DLBCL, underscoring the educational significance for clinicians. It highlights the importance of recognizing atypical presentations of PS-DLBCL, such as the rare initial symptoms of neck and shoulder pain observed in this patient, which can greatly enhance our understanding of the disease and improve diagnostic accuracy. The rarity of this presentation and its potential to mimic more common conditions make it a valuable case study for medical education and clinical practice (3).

## 2 Case description

**Figure 1:**
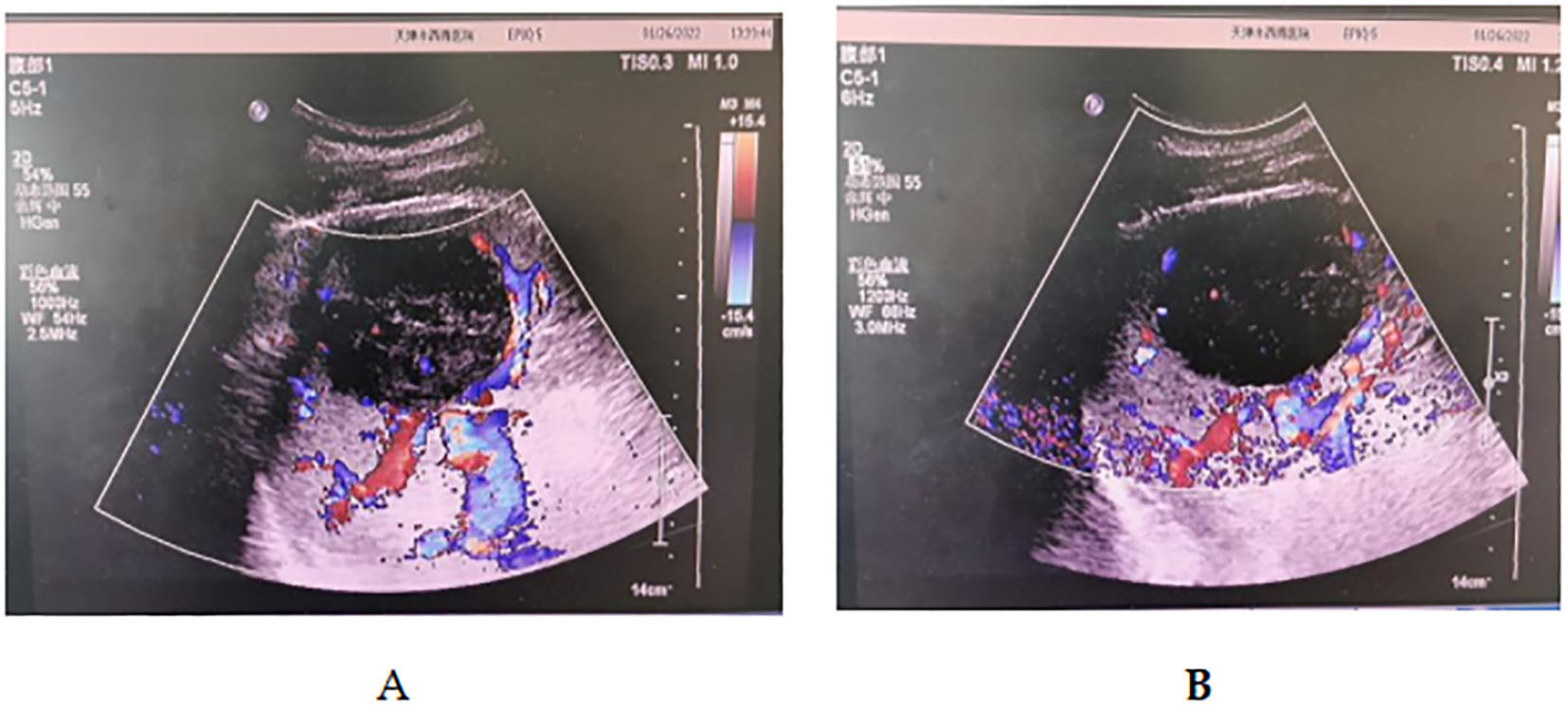
**(A, B)** Ultrasound imaging revealed a 7.8 x 6 cm hypoechoic mass within the spleen.

A man aged 31, who had been healthy before, arrived at the emergency room with neck and left upper shoulder pain. The patient reported a gradual worsening of these symptoms over the preceding 12 hours, without any other associated complaints. There were no indications of respiratory distress, gastrointestinal symptoms, night sweats, initial abdominal pain, or history of specific trauma. The patient was asymptomatic for unexplained fever, persistent fatigue, and weight loss. There were no reported symptoms of joint pain, swelling, or stiffness. The patient denied having an autoimmune disease or a family history of connective tissue disorders. The patient’s vital signs included a body temperature of 36.5°C, heart rate of 110 beats per minute, blood pressure of 130/80 mmHg, respiratory rate of 19 breaths per minute, and oxygen saturation in room air of 95%.On physical examination, the patient was alert, awake, and oriented but visibly uncomfortable. Bilateral lung fields were clear, with no abnormal heart rate or rhythm detected. The abdomen was not distended and was soft, but no tenderness was noted upon comprehensive abdominal examination. Lab tests showed hemoglobin levels at 14.1 g/dL, white blood cell count at 11.39 x 10^9/L, neutrophils at 9.11 x 10^9/L, neutrophil percentage at 79.9%, and platelet count was normal. There were no evident abnormalities in liver, kidney, coagulation profiles, serum electrolytes, markers of pancreatitis, or cardiac enzymes. Infectious disease-related tests, including those for hepatitis A, B, C, and HIV, were negative. FAST imaging revealed intraperitoneal and perisplenic fluid, along with splenic masses, resulting in the diagnosis of splenic rupture (**Figure 1**). Serological tests for EBV were negative, and there was no clinical or histological evidence to suggest an association with EBV.

**Figure 2:**
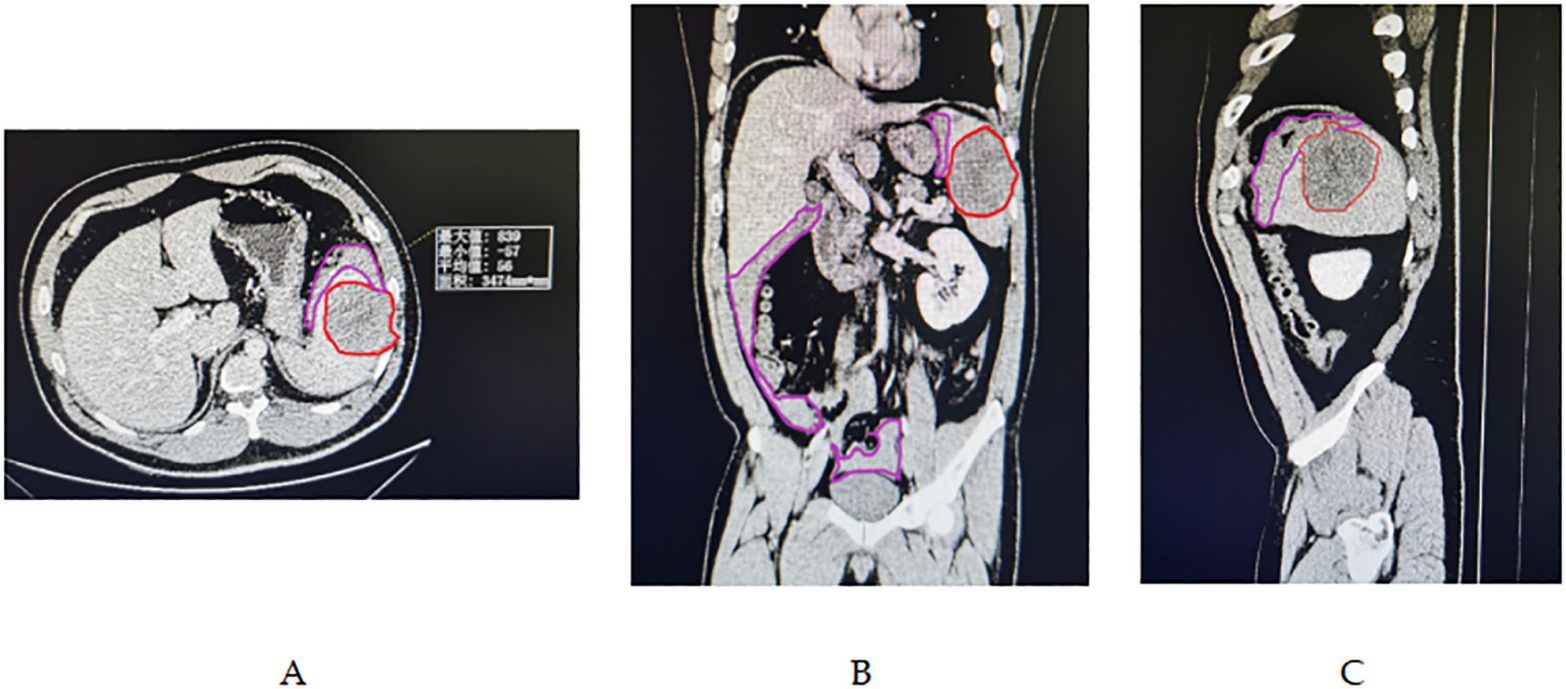
**(A-C)** Abdominal enhanced CT imaging revealed a large splenic mass, demarcated by the red line, measuring 7.8 x 6.5 cm in diameter, situated within a homogeneous low-density area. This mass was well-separated from the surrounding healthy splenic tissue. Additionally, a substantial amount of hemoperitoneum was detected within the pelvic, perisplenic, and perihepatic regions, as indicated by the purple line demarcating the area.

**Figure 3:**
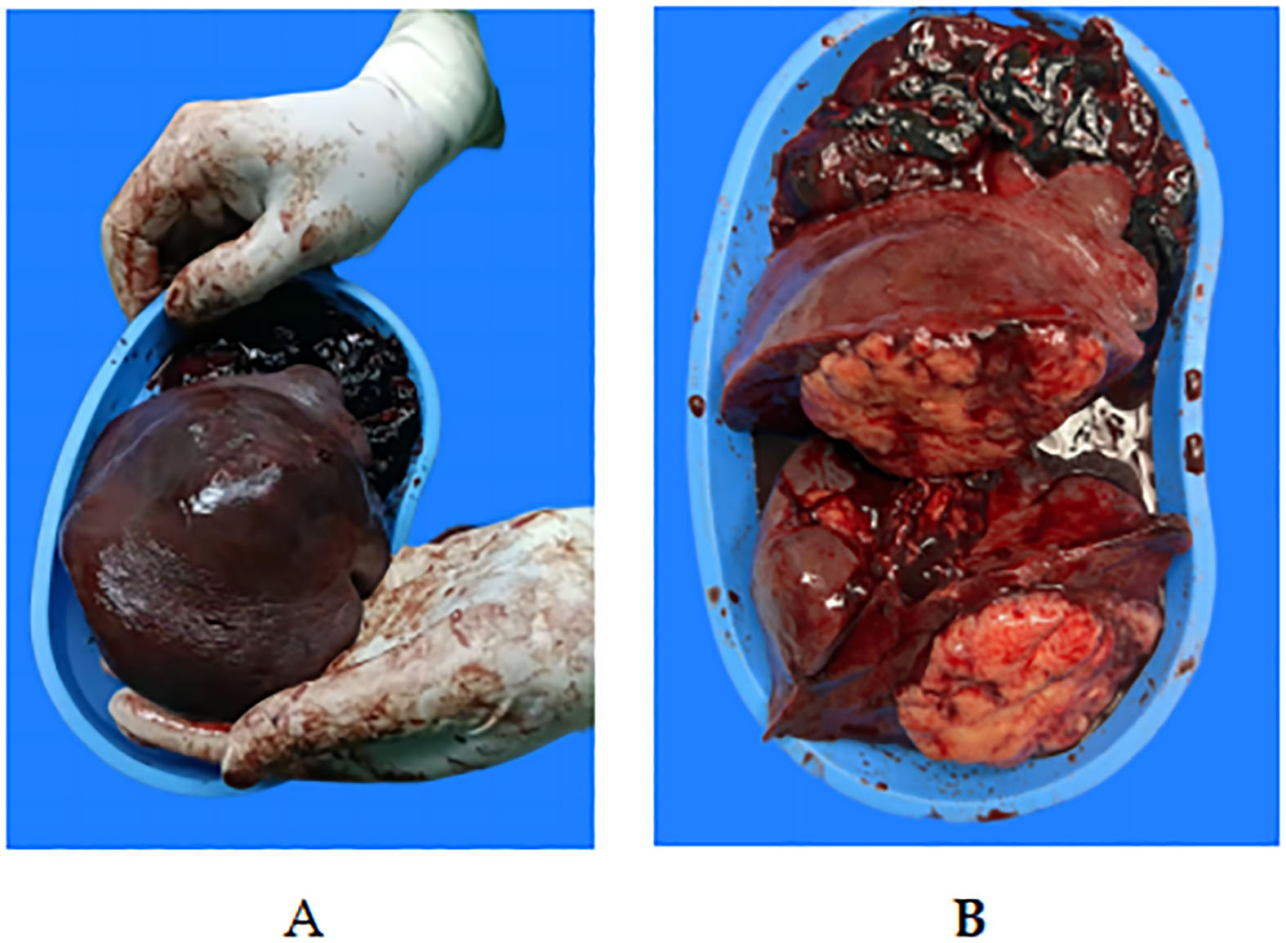
Macroscopic view of the surgical specimen.**(A, B)** Gross specimen: Macroscopy revealed a bulging splenic cut surface. The intrasplenic tumor mass had a yellow macroscopic appearance, with polycyclic contours crossed by fibrous partitions. These nodules were clearly separated from the normal spleen parenchyma. A tear of 2 cm was observed on the capsule of the splenic tumor.

**Figure 4:**
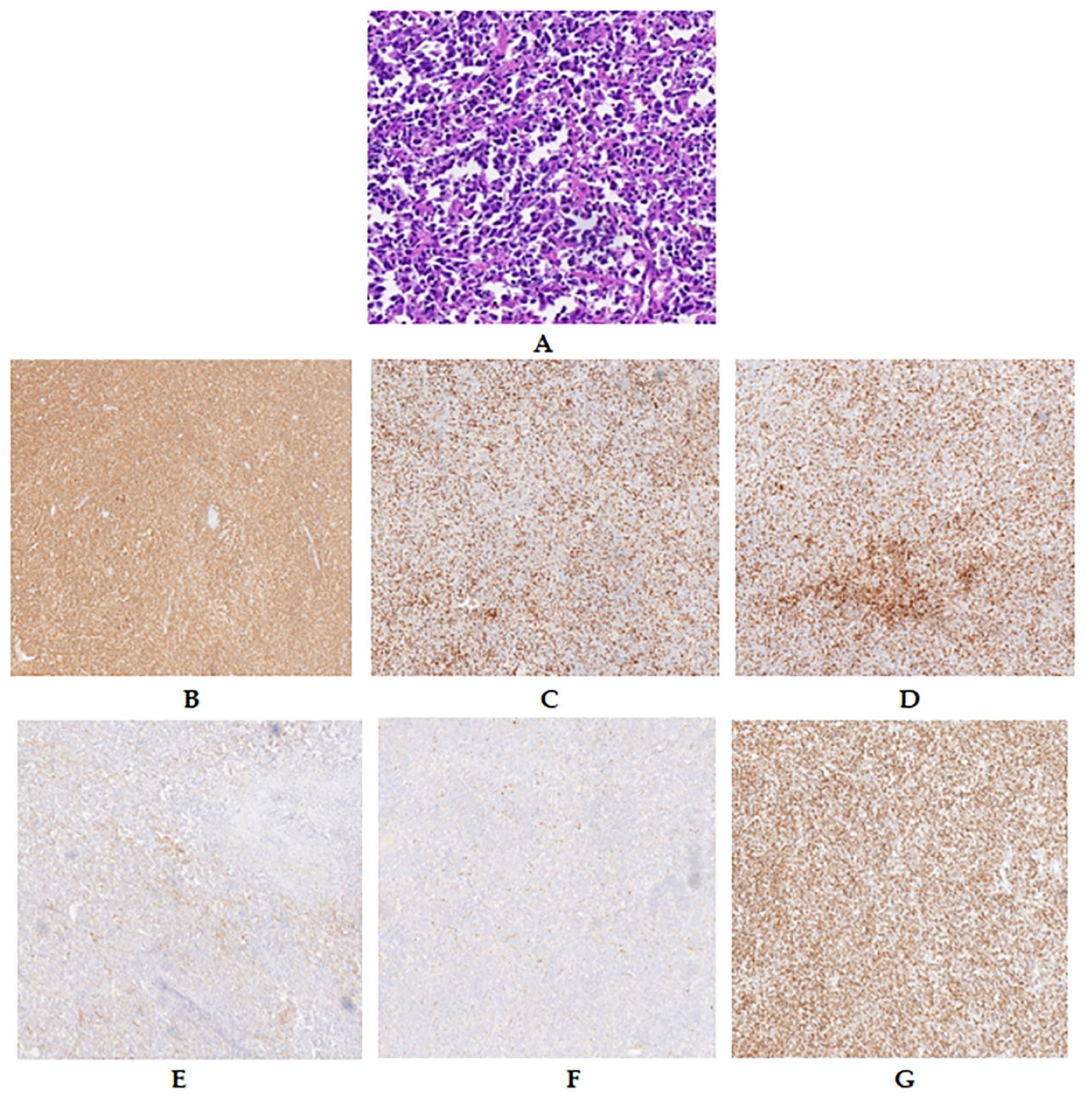
Pathological features of the spleen: lymphomatous cells filling the splenic sinuses **(A)**. IHC features of the spleen. Diffuse and strong CD 20 positivity was observed in lymphoma cells **(B)**. Lymphomatous cells were weakly positive for CD 3 **(C)**, CD 5 **(D)**, and CD 10 **(E)** but negative for Cyclin-d 1 **(F)**. The Ki-67 proliferation index was approximately 80% **(G)**.

In the emergency room, the patient’s activity was restricted, and he was subjected to ECG monitoring, analgesia, initial intravenous (IV) fluid resuscitation, and high-flow oxygen therapy. Initially, the patient was diagnosed with an unexpected rupture of the spleen and quickly moved to the general surgery ward. Within 24 hours of hospitalization, a contrast-enhanced CT scan was performed, which revealed splenic rupture and an intrasplenic mass shadow (diameter, 8 cm) with a clear boundary, suggestive of lymphoma origin. Significant amounts of pelvic, perisplenic, and perihepatic fluids were also noted (**Figure 2**). The radiological results suggested that the spleen had ruptured spontaneously. The individual experienced hemodynamic instability, resulting in a decrease in hemoglobin levels from 141 g/L to 80 g/L. Subsequently, a second ultrasonic examination revealed a significant increase in hydrops around the spleen. Consequently, an emergency laparotomy was immediately scheduled.1500 cc of blood was removed from the abdominal cavity during the procedure. The findings included bleeding and splenic rupture in the upper left quadrant, with an 8 cm mass observed in the posterior part of the splenic hilum. The splenic capsule in the mass spontaneously ruptured with active bleeding (**Figure 3**). The patient underwent splenectomy, during which spontaneous splenic rupture was confirmed. The histological analysis supported the identification of diffuse large B-cell lymphoma (DLBCL). Immunohistochemical (IHC) staining revealed positive staining for CD 20 (diffuse), CD 3, CD 10, CD 5, CD 68, CD 31, CD 34, and CD 56 (some cells), while CD 30, Cyclin-D 1, and CD 138 were negative. Additionally, the Ki67 proliferation index was found to be 80% (**Figure 4**). The patient had a smooth recovery after surgery and was released from the hospital after a 10-day stay.

Prior to the operation, no other diseases were identified through chest/abdomen/pelvis CT scans. Additionally, PET imaging did not reveal any residual lymphoma post-operation. The final diagnosis of primary splenic diffuse large B-cell lymphoma (PS-DLBCL) was made based on the results of the pathological examination, as well as imaging and immunohistochemical characteristics. The patient underwent six cycles of R-CHOP chemotherapy in conjunction with postoperative chemotherapy. To date, there have been no abnormal findings during follow-up spanning nearly six months.

## 3 Discussion

This report details the case of a man with primary splenic diffuse large B-cell lymphoma (PS-DLBCL) who suffered a spontaneous splenic rupture but was able to recover thanks to prompt diagnosis and surgical treatment. Splenic rupture is widely acknowledged as an uncommon and serious issue, marked by spleen injury causing internal bleeding. As per the guidelines set by Orloff and Peskinin in 1958, splenic rupture is classified as ‘spontaneous’ only when it happens without any prior trauma or spleen conditions (4). 1% of splenic ruptures are due to spontaneous causes, resulting in a mortality rate of 10–15%.Ninety-three percent of spontaneous splenic ruptures (SSRs) are associated with a histopathologically altered spleen (5). Most cases of SSR are attributed to three main conditions: hematologic cancer (30.3%), infectious illnesses (27.3%), and inflammatory or neoplastic disorders (20.0%) (6).

Hematological cancers often involve primary splenic lymphoma (PSL), which is identified by a tumor limited to the hilar lymph nodes and spleen, making up only 1%-2% of lymphoma cases (7, 8). This subset was first described by Das Gupta and colleagues in 1965 (9). The suggested guidelines for identifying primary splenic lymphoma involved identifying splenomegaly and compression of nearby organs as the initial symptoms. During splenectomy, the tumor was found to be limited to the lymph nodes of the spleen and hilum, with no signs of spreading to other areas. No other regional lymphoma should have developed six months post-diagnosis. Ahmann et al. Implemented the Ahmann staging method, which classified stage I cancers as those limited to the spleen without affecting the splenic lymph nodes or distant organs (10). Stage II tumors affected the lymph nodes near the spleen; Stage III tumors spread to the liver, lymph nodes in the abdomen, and bone marrow in distant locations.

Despite the spleen being involved in 50% of non-Hodgkin lymphoma (NHL) cases, PS-DLBCL remains a rare subcategory of PSL, with an incidence of less than 1% among all NHLs (11, 12). Common findings in the physical examination of PS-DLBCL cases include splenomegaly accompanied by mass lesions, impairment of performance status, abdominal pain, and B symptoms (3). In some cases, an advanced extranodal lesion may also be present at the time of admission (13). However, spontaneous splenic rupture is a rare complication of PS-DLBCL, with one study reporting a rate of 0.2% among malignant lymphomas (4). Primary central nervous system lymphoma demonstrates the most aggressive behavior among non-Hodgkin lymphomas, especially when accompanied by splenic rupture, posing a significant risk to life if not addressed promptly. The exact cause of splenic rupture in cases of blood cancer has not been firmly determined (14). Some suggested reasons include the physical impact of lymphoma cells infiltrating the spleen, particularly when the outer layer is breached, bleeding beneath the outer layer, rupture of the outer layer due to spleen tissue death, and irregular blood clotting (15). These mechanisms may act synergistically to cause splenic rupture in hematological malignancies.

Although splenic rupture is not commonly encountered in hematological diseases, mortality associated with this condition can be as high as 21% in cases where neoplastic disease is present (16). Clinicians, whether hematologists or emergency physicians, must be cognizant of this uncommon complication, as it can significantly elevate the risk of mortality. The symptoms of patients with splenic rupture can vary. It is frequently reported that abdominal pain occurs when the spleen ruptures (17). Patients with minor injuries may exhibit abdominal pain, upper abdominal tenderness, and left upper abdominal discomfort (18). During the abdominal assessment, indications of peritonitis, Kehr’s sign, and the Balance sign may be detected. Additional symptoms that may be present are nausea, vomiting, or episodes of syncope.Kehr’s sign, indicating severe radiation pain, was observed in the left shoulder in 20% of instances. Signs of hypovolemic shock are frequently observed in instances of more severe splenic injuries (19). Hence, it is important to consider the possibility of splenic rupture in any patient experiencing sudden left upper abdominal pain, persistent hemodynamic instability, or acute anemia (20). However, the sole clinical clue may be the patient’s early shoulder and neck pain, which could be caused by a small amount of hemoperitoneum stimulating the phrenic nerve (21). Furthermore, it was only when the patient’s hemoglobin level significantly decreased and they entered a state of shock that upper abdominal pain became apparent.

In fact, the choice of treatment strategy for spontaneous splenic rupture (SSR) is contingent upon hemodynamic stability and the underlying pathology. It is of utmost importance for imaging examinations and hemodynamic monitoring to provide actionable data for the diagnosis and treatment of SSR. FAST was the main imaging technique utilized to confirm the diagnosis of SSR and was quickly and safely performed in the emergency department. The CT examination demonstrated a specificity and sensitivity of over 95% in detecting splenic disease, and it is considered a cost-effective means of addressing specific questions, which delineates the necessity of specific interventions, the pathogenesis of splenic lesions, and their severity (22). The integrity of the splenic pulp must be confirmed through CT and ultrasound, while hematocrit and hemoglobin levels should be determined at 4–6-hour intervals within the initial 24 hours until the patient’s condition stabilizes (23, 24). The conservative method is rarely employed in the diagnosis of SSR, as selecting patients who may benefit from conservative surgical treatment remains a subject of debate.Patients with stable hemodynamics can be treated conservatively, including bed rest, monitoring, a series of abdominal examinations, and blood and fluid administration as required. For patients with hemorrhagic shock caused by hemodynamic instability, splenectomy should be the first-line life-saving treatment.

The treatment of isolated splenic lesions, particularly in the context of localized DLBCL, often involves splenectomy, which has been shown to improve disease-free survival (DFS) and overall survival (OS) (25). Recent studies have highlighted the heterogeneity of DLBCL at the phenotypic and genetic levels, leading to a more nuanced understanding of treatment strategies (25). The impact of splenectomy on prognosis has been a subject of extensive research, with some studies suggesting improved outcomes following splenectomy in terms of DFS and OS, especially in cases of isolated splenic involvement in lymphoma (26).

Typically, risk factors for death-related SSR include splenomegaly, neoplastic diseases, and age over 40 years (2). Additionally, research has indicated that complete removal of the spleen is done in cases of tumors causing spontaneous splenic rupture (27). Given our patient experienced a decrease in hemoglobin levels and shock during the 24-hour observation period, emergency splenectomy was chosen as a life-saving intervention. If the patient has stable circulation and minimal blood loss, laparoscopic splenectomy is also safe and feasible. A confirmed diagnosis of primary splenic diffuse large B-cell lymphoma (PS-DLBCL) cannot be determined without obtaining a gross specimen through surgery, as pathological examination is considered the most reliable method for diagnosis (28). In the end, the examination of the spleen showed it to be in line with diffuse large B-cell lymphoma (DLBCL). Combined with pre- and postsurgical imaging and immunohistochemical features, the final diagnosis was PS-DLBCL. In our patient, early diagnosis with an appropriate therapeutic strategy for spontaneous splenic rupture was challenging due to nonspecific symptoms. Diagnosing this disease presents a challenge for clinicians. Imaging and blood analysis are crucial in managing acute abdominal conditions. Detecting free fluid in the abdomen and monitoring changes in shock indices are important factors in assessing the risk of splenic rupture. For primary splenic lymphoma, there is no standardized diagnosis and treatment plan. However, emergency splenectomy is the only viable treatment for PS-DLBCL complicated by splenic rupture, and postoperative chemotherapy, such as the R-CHOP regimen (3), is necessary. Chemotherapy and radiotherapy tailored to the pathological conditions after surgery can enhance patient survival rates.

## 4 Conclusion

This report presents a rare case of PS-DLBCL in which the patient experienced spontaneous splenic rupture accompanied by neck and shoulder pain. This case underscores the importance of aggressive imaging assessments for patients presenting with atypical symptoms, such as neck and shoulder pain, which may be indicative of an underlying splenic issue. The decision to perform a splenectomy should be considered earlier in the treatment algorithm for such patients, given the potential life-threatening complications of a ruptured spleen. Early diagnosis relies on imaging studies such as ultrasound and CT scans. Treatment should be tailored according to the patient’s hemodynamic status and the severity of splenic lesions, with emergency splenectomy being considered when necessary. Following diagnosis, standard pathological examination and immunohistochemical analysis should be conducted, and subsequent treatment plans should be formulated based on the patient’s condition.

## Data Availability

The raw data supporting the conclusions of this article will be made available by the authors, without undue reservation.
